# Extrafloral nectary-bearing plant *Mallotus japonicus* uses different types of extrafloral nectaries to establish effective defense by ants

**DOI:** 10.1007/s10265-019-01119-5

**Published:** 2019-06-21

**Authors:** Akira Yamawo, Nobuhiko Suzuki, Jun Tagawa

**Affiliations:** 1 Department of Applied Biological Sciences, Faculty of Agriculture, Saga University, Saga, 840-8502 Japan; 2 Department of Biosphere–Geosphere System Science, Faculty of Informatics, Okayama University of Science, Okayama, 700-0005 Japan

**Keywords:** Ant–plant mutualism, Biotic defense, EFN, Induced defense, Leaf damage

## Abstract

Extrafloral nectary (EFN)-bearing plants attract ants to gain protection against herbivores. Some EFN-bearing plants possess different types of EFNs, which might have different effects on ants on the plants. *Mallotus japonicus* (Thunb.) Muell. Arg. (Euphorbiaceae) bears two types of EFNs, including a pair of large EFNs at the leaf base and many small EFNs along the leaf edge. This study aimed to determine the different roles of the two types of EFNs in biotic defense by ants. A field experiment was conducted to investigate the effect of leaf damage on EFN production and on the distribution pattern of ants. After leaf damage, the number of leaf edge EFNs increased in the leaves first-produced. The number of ants on the leaves also increased, and the foraging area of ants extended from the leaf base to the leaf tip. An EFN-covering field experiment revealed that leaf edge EFNs had a greater effect than leaf base EFNs on ant dispersal on leaves. The extended foraging area of ants resulted in an increase of encounter or attack rate against an experimentally placed herbivore, *Spodoptera litura*. These results suggest that *M. japonicus* plants control the foraging area of ants on their leaves using different types of EFNs in response to leaf damage, thus achieving a very effective biotic defense against herbivores by ants.

**Electronic supplementary material:**

The online version of this article (10.1007/s10265-019-01119-5) contains supplementary material, which is available to authorized users.

## Introduction

Mutualism is an interaction between different species that enhances the fitness of both partners involved. Types of mutualism include exchanging resources, services, and services for resources (Doebeli and Knowlton 1998; Leigh 2010). Mutualisms often exhibit a delicate balance between the costs and benefits for each species involved (Bronstein 1998; Ferriere et al. 2002). Because the costs and benefits in mutualisms often depend on the behaviors of the involved partners, organisms sometimes control the behavior of their partners to increase their own benefits (Heil et al. 2014; Suzuki and Ohashi 2014; Vander Wall 2010; Wright et al. 2013).

In a type of plant–animal mutualism involving the exchange of resources for services, plants provide nectar [floral and extrafloral (EF) nectar] as a food resource to pollinator partners (Armbruster 1993) and to predatory ant partners capable of deterring the plant’s natural enemies, such as herbivorous insects (Bronstein 1998; Koptur 1992). In such a type of mutualistic system, plants have evolved mechanisms to control the behavior of their partners to increase their own benefits (reviewed in Grasso et al. 2015). Some extrafloral nectary (EFN)-bearing plants can control the foraging behavior of ants through nectar quality (Heil et al. 2014; Wilder and Eubanks 2010). *Acacia* trees have evolved an obligate ant–plant mutualism by secreting chitinase-containing EF-nectar, which inhibits the sucrose hydrolytic activity in ant midguts. Therefore, ant partners depend on *Acacia* trees that secrete sucrose-free EF-nectar (Heil et al. 2014).

In addition to changing the quality of EF nectar, EFN-bearing plants can alter the quantity of nectar or developmental patterns of reward-providing organs (e.g. Grasso et al. 2015; Ness 2003). Several studies report that EFN-bearing plants regulate the number of EFNs in response to abiotic and biotic factors (Ness 2003; Pulice and Packer 2008; Wooley et al. 2007; Yamawo et al. 2014, 2015), consequently controlling the number of ants on the plants and thus enhancing the efficacy of defense by ants (Ness 2003; Yamawo et al. 2014). Some plant species secrete large amounts of EF nectar from young or middle-aged leaves and less nectar from old leaves (Radhika et al. 2008; Yamawo et al. 2012b), attracting numerous ants to important parts for their growth.

**Fig. 1:**
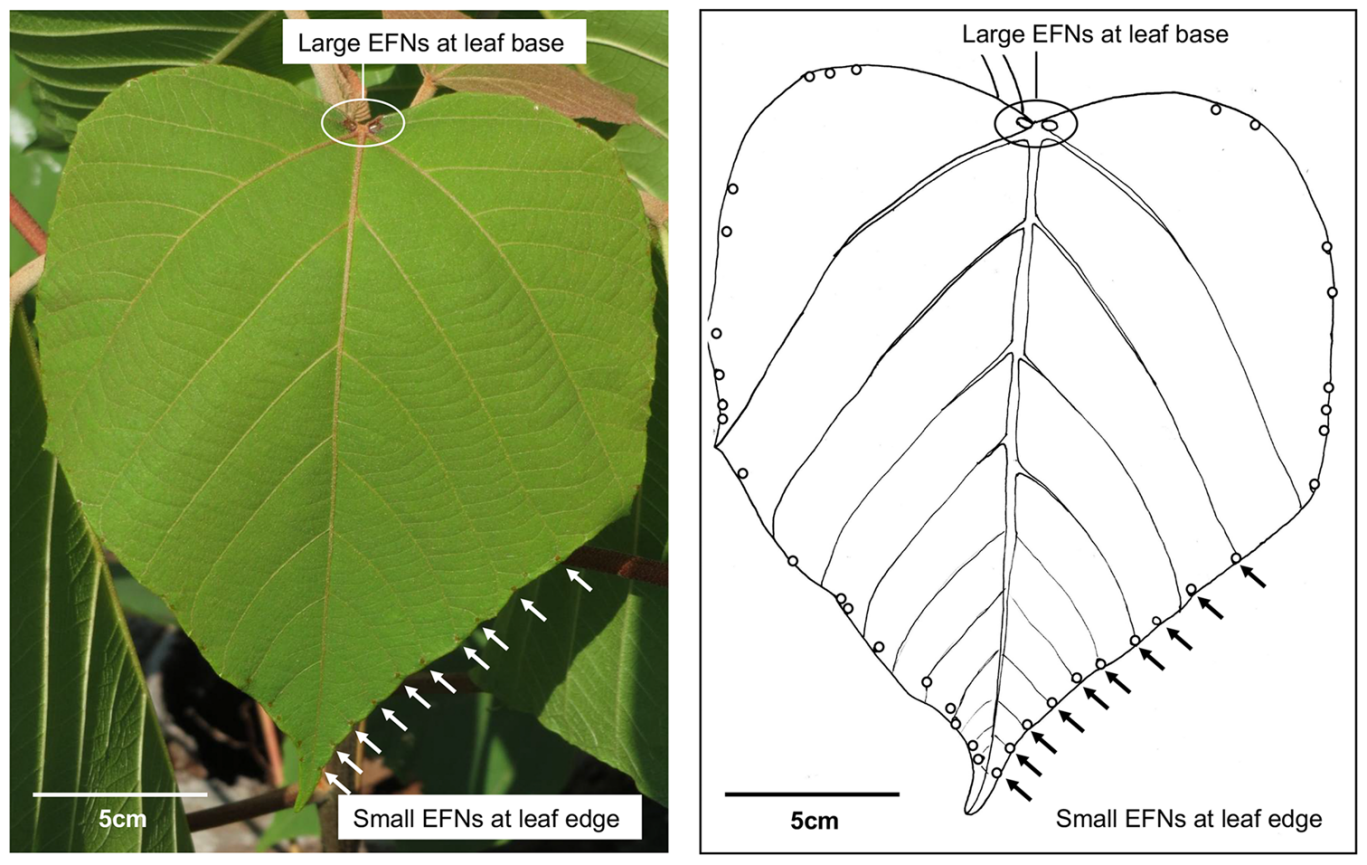
Two types of extrafloral nectaries (EFNs) occur on the leaves of *Mallotus japonicus*. A pair of large EFNs is located at the leaf base and small EFNs are scattered along the leaf edge. Arrows represent some of the many edge EFNs

*Mallotus japonicus* (Thunb.) Muell. Arg. (Euphorbiaceae) has two types of EFNs, differing in size and number, including a pair of large EFNs at the leaf base and small EFNs of indefinite number along the leaf edge (Yamawo et al. 2012a) (Fig. 1). The edge EFNs are variable in number and are inducible in response to leaf damage (Yamawo and Suzuki 2018). This induction is regarded as an adaptive response to herbivory because an increased number of EFNs would augment the ant-attractive ability of the plants and, in turn, reduce leaf damage by herbivores (Yamawo et al. 2012a, b, 2014). However, this phenomenon cannot explain the existence of multiple types of EFNs. For plant defense, the attracted ants should walk around the leaf surfaces. Therefore, if a plant with “ants” is severely damaged, the plant should scatter ants more widely on its leaves. We hypothesize that the fundamental role of the large EFNs is to attract ants to the leaves and that of the small EFNs, in addition to attracting ants, is to scatter or disperse the ants on the leaf surfaces because the small EFNs are located along the leaf edge, from the leaf base to the tip. This study aimed to determine the different roles of the two types of EFNs in biotic defense by ants in *M. japonicus*.

## Materials and methods

### Study species

*Mallotus japonicus* is a pioneer plant that grows in the canopy gaps and disturbed areas of forests in the temperate regions of eastern Asia. The plant bears a pair of large EFNs at the leaf base and small EFNs of indefinite number (0 to ~ 50) along the leaf edge (Fig. 1) (Yamawo et al. 2012a). Leaf base EFNs are located along the central vein, whereas the edge EFNs are not. EFNs function as an indirect defensive trait by attracting many ant workers of various species (Yamawo et al. 2014).

### EFN production in response to leaf damage

During Sep–Oct in 2009, 100 seeds of *M. japonicus* were collected from 10 trees growing in the Okayama Prefecture, western Japan (33°41′N, 133°55′E). On 02-Mar-2010, a plastic container (45 cm × 35 cm × 15 cm) was filled with wet red soil to a 5-cm depth. The collected seeds were then sown at a 1-cm depth. The container was incubated in a growth chamber (Biotron; NK System, Osaka, Japan) at 35 °C under a 12L–12D photoperiod for 24 h because *M. japonicus* seeds germinate after exposure to high temperatures (Washitani and Takenaka 1987). Thereafter, the container was maintained in the growth chamber at 25 °C under the same photoperiod for 30 days. The container was watered every other day. On 01-Apr-2010, 45 healthy seedlings with two cotyledons and one leaf were selected for the experiment. The seedlings that had reached approximately 4-cm height were individually transplanted into plastic pots (20 cm × 20 cm × 25 cm) containing 70% tuff loam and 30% humus. The pots were placed in a greenhouse at Saga University (33°24′N, 130°29′E). The pots that were sufficiently watered on alternate days (2 l per plant) were cultivated for 108 days in the greenhouse to prevent any invasion by herbivorous insects. On 18-Jul, 21 of the 45 pots were randomly assigned to a leaf-damage treatment, and the remaining 24 pots were designated as controls. All the pots were placed in an experimental field at Saga University. Control and treatment pots were alternately installed. All plants were approximately 25 cm in height and bore approximately 10 leaves. Prior to the leaf-damage treatment, the number of edge EFNs on the fully expanded fifth leaves of both pretreatment and control plants were counted.

The leaf damage treatment was performed on 21-Jul-2010. For treated seedlings (*n* = 21), the distal half of every leaf was removed using scissors. Young *M. japonicus* plants are usually damaged at a level of 10–80% by the inchworm *Ascotis selenaria* (Lepidoptera: Geometridae) under natural field conditions (Yamawo et al. 2012b). Hence, we selected a damage level of 50%. The controls (*n* = 24) were undamaged. After 30 days, to determine the effect of the treatment on EFN production, we counted the number of base and edge EFNs on the leaves first-produced after the treatment. On the day of counting, these leaves were positioned proximate the fourth from the apex and were fully expanded. The surface of the target leaf of each plant was photographed using a digital camera (IXY Digital 810 IS, Canon, Tokyo) during 9:00–12:00 when the ant activity was high (Yamawo et al. 2012b). To determine the ant positions on the leaf, the leaf area was divided into 10 segments, equally-spaced from the base to the tip, and then we counted the number of ant workers, which were later identified in our laboratory, on each segment. For each target leaf, the coefficient of variation (CV) in the relative positions of ants from the leaf base was calculated to express the variation of ant distributions within a leaf.

### Nectar volume and sugar concentration

During Sep–Oct in 2012, 20 seeds of *M. japonicus* were collected from five trees growing in Okayama. On 02-Apr-2013, a plastic container (45 cm × 35 cm × 15 cm) was filled with wet germ-free soil to a 10-cm depth, and the seeds were sown at a 1-cm depth. This container was incubated in the same way as described above and maintained at 25 °C under a 12L–12D photoperiod for 1 month. The plants were watered every other day. On 01-May-2013, 20 plants were individually transplanted into plastic pots as mentioned above. These plants were placed in the experimental field at Saga University and cultivated for 3 months.

On 30-Jul-2013, the plants, which now had approximately eight leaves, were transferred to the greenhouse, which was free of nectar-collecting insects. To determine the activity of EFNs, the third leaves from the apex of the plants were examined because the nectar secretion of the middle-aged leaves was active (Yamawo et al. 2012b). The nectar secreted from each EFN type was collected. Before the nectar collection, all leaves were carefully washed with distilled water to remove any residual nectar on the leaves. After a 24-h period, newly-secreted EF nectar was collected from every EFN using 0.5-µl microcapillary tubes, and nectar volume was optically measured. Sugar concentration in the nectar was immediately measured after the collection using a portable temperature-compensated refractometer (ATAGO hand refractometer, L. Kubler, Karlsruhe, Germany).

### EFN utilization by ants

Field surveys were conducted at Mt. Kinryu (33°33′N, 130°31′E; altitude, 40–250 m) in Kanzaki City, Saga Prefecture, in western Japan. The mean ± SD annual precipitation in Saga during the last decade (2004–2013) was 1869 ± 249 mm. The corresponding mean annual air temperature was 16.9 ± 0.3 °C (Japan Meteorological Agency, http://www.data.jma.go.jp/obd/stats/etrn/).

To examine whether the two EFN types affected ant behavior differently, the nectar-sucking time of ants on each EFN type was measured in the field. On 14-Sep-2013, 20 plants of 30–50-cm height, bearing low levels of leaf damage (< 3%), were randomly selected. The third leaves of the selected plants were then observed during 9:00–12:00. These leaves attracted one of two ant species, including *Pheidole noda* Smith (*n* = 15) and *Crematogaster teranishii* Santschi (*n* = 5). The efficacies of biotic defense by these two ant species on *M. japonicus* have been reported to be high (Yamawo et al. 2017). For each EFN type on the target leaf of each plant, the nectar-sucking time of one ant worker (randomly selected) was recorded once during the observation period.

### Roles of two EFN types in biotic defense

To determine the roles of two EFN types in indirect defense or ant attraction, an EFN-covering experiment was conducted in the field using varnish containing urethane resin (Asahipen, Tokyo, Japan) as an EFN-covering material. During 9:00–12:00 on 15-Sep-2013, 98 young *M. japonicus* plants of 40–50 cm height, growing at the forest edges on Mt. Kinryu, were randomly selected. The third fully expanded leaf from the apex of each plant was used for the experiments. The mean ± SD number of the edge EFNs on the third leaf was 9.8 ± 6.3 (*n* = 98) before the treatment was applied. The selected 98 plants were assigned to the following five treatments: (1) leaf base EFNs functioning (all edge EFNs covered, *n* = 22); (2) all edge EFNs functioning (leaf base EFNs covered, *n* = 22); (3) four edge EFNs functioning (all EFNs except for two edge-centered EFNs and two leaf tip EFNs covered, *n* = 22); (4) no EFNs functioning (all EFNs covered, *n* = 10); and (5) control (no EFNs covered, *n* = 22). The plants with four edge EFNs functioning were prepared to nullify the effect of EF nectar volume on ant attraction. The nectar volumes secreted by the four edge EFNs were approximately similar to those secreted by the leaf base EFNs (refer to Results). A drop (ca. 1–2 µl) of varnish was applied to each EFN to cover them. Control leaves received a varnish application at 10 places on the leaf edge, avoiding EFNs. The cover of particular EFNs did not influence in secretion of other EFNs (Akira Yamawo unpublished data). During 9:00–12:00 on the following day, the third-leaf surfaces of the plants were photographed using a digital camera. The number of ants in each photo was then counted, and the ant positions on the leaf were assessed in the same manner as described above.

On young *M. japonicus* plants in the field, *Spodoptera* spp. caterpillars were occasionally observed consuming leaves (Yamawo et al. 2014). Therefore, on each treated plant, a single 6th-instar caterpillar of *Spodoptera litura* was placed as a herbivore at the center of the third leaf from the apex during 9:00–12:00. To estimate the intensity of biotic defense induced under each treatment, the ants’ attack on the caterpillar at the time of the first encounter was observed. Here, the attack was defined as biting. The caterpillar was removed from the leaf after an encounter or after 30 min of being on the leaf. Then, the encounter and attack rates on the leaves of each EFN-treatment condition were calculated. The attack rate was defined as the proportion of attack events to encounter events.

### Statistical analyses

Data were analyzed using R v.2.15.1 software (R Development 2012). The numbers of two EFN types on leaf-damaged and undamaged plants were analyzed using a generalized linear model (GLM) with a negative binomial distribution and log link function. Treatment (leaf-damaged or undamaged), EFN types (leaf base or leaf edge), time (pre- or post-treatment), and their interactions were included as explanatory variables. When these interactions were significant, the effects of the treatment on the numbers of each EFN type were analyzed.

The number of ant workers, their relative position within a leaf, and the CV of the relative position of ant workers were also compared between leaf-damaged and undamaged plants using a GLM with a Poisson distribution and a log link function. To compare the ant species composition between leaf-damaged and undamaged plants, Bray–Curtis dissimilarity matrices were calculated, followed by an analysis of similarity (ANOSIM).

The EF nectar volume and the sugar concentration of the EF nectar were compared between the EFN types using a *t* test. Nectar-sucking times by ants were compared between the two EFN types using a generalized linear mixed model (GLMM) with a negative binomial distribution and log link function. Plant ID was included as a random effect in this model.

For the comparison of ant species composition among the different EFN treatment leaves, Bray–Curtis dissimilarity matrices were calculated, followed by ANOSIM. *P* values were subsequently adjusted by Holm’s sequential correction method. Ant abundance, the relative position of ant workers on a leaf, and the latter’s CV were separately compared among the five treatments using a GLM with a negative binomial distribution and log link function. Multiple comparisons of the treatment means were performed using Steel–Dwass test. However, the relative position of the no EFNs treatment was excluded from the analysis because of a lack of ant visits. In addition, CVs of the four edge EFNs and no EFNs treatments could not be calculated because of a lack of ant visits.

The encounter rates of ants with *S. litura* caterpillars and their attack rates were compared among the EFN treatments using Fisher’s exact test. *P*-values were corrected using Holm’s method. The association between the attack rate of ants against herbivores and the ant abundance, the relative position of ant workers, or the CV of relative position of ant workers was all separately examined using a GLM with a binomial distribution and a logit link function. We conducted the likelihood ratio test in GLM and GLMM analyses.

## Results

### EFN production in response to leaf damage and the ant foraging area

**Fig. 2:**
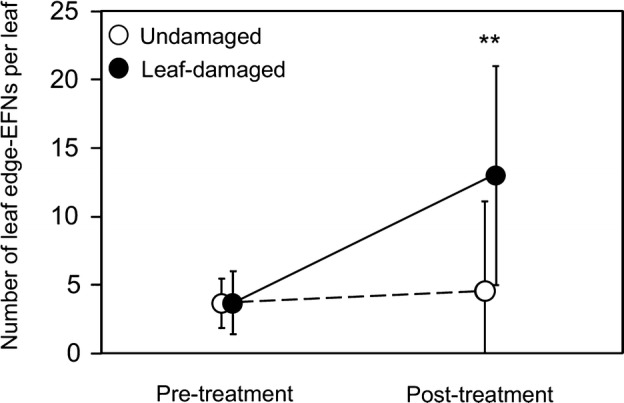
Leaf-damage effect on EFN production in the first-produced leaf of *Mallotus japonicus* after treatment (refer to “Materials and methods” for details). EFNs of the pretreatment plants were those of the fifth leaves. Leaf-damaged, *n* = 21; undamaged, *n* = 24. Bars represent mean ± SD. **Significantly different (GLM, *P* < 0.01)

The effects of leaf damage on EFNs differed between their types (Table S1). The number of the leaf base EFNs was consistently two for both the undamaged and leaf-damaged plants (Table S2). However, the number of edge EFNs in the leaf-damaged plants was significantly larger than that in the undamaged plants (Fig. 2; Table S3). Prior to leaf-damage, the number of edge EFNs of the treated plants did not differ from that of the untreated plants (Table S3). The effect of leaf damage on EFN production was obviously restricted to the edge EFNs (Table S2).

**Fig. 3:**
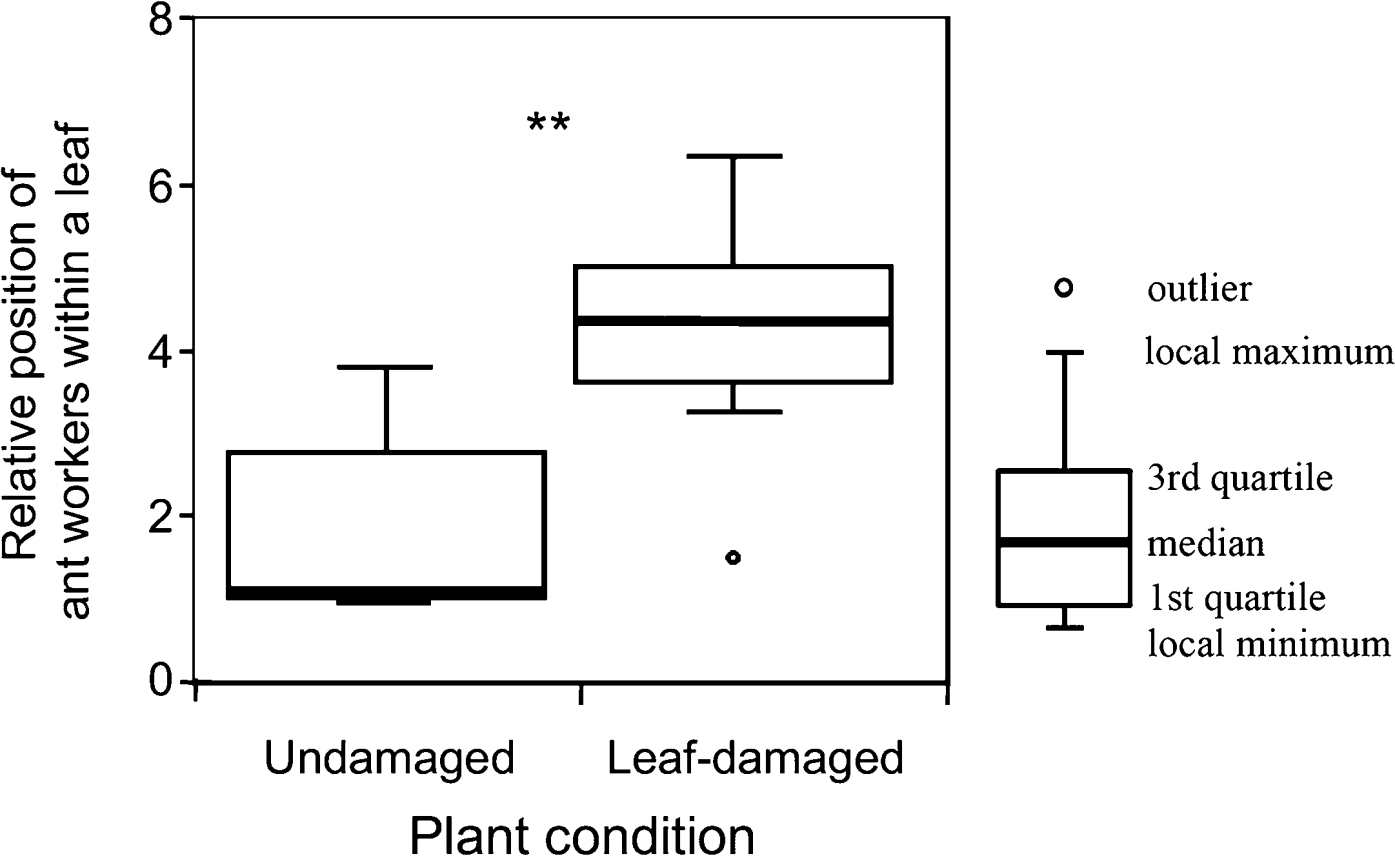
Relative position of the ant workers on the first-produced leaf of leaf-damaged (*n* = 21) and undamaged (*n* = 24) *Mallotus japonicus* (refer to “Materials and methods” for details). **Significantly different (GLM, *P* < 0.01)

In the present study, at least one of following three ant species was found on each plant: *P.* *noda* (22/45), *Pheidole indica* Mayr (15/45), and *Nylanderia flavipes* Smith (7/45). The species composition of ants on the plants did not differ between the treatments (ANOSIM, global *R* = 1.33, *P* = 0.99). The number of ant workers on the leaf-damaged plants was greater than that on the undamaged plants [undamaged, 2.54 ± 1.41 (mean ± SD), *n* = 24 and leaf-damaged, 5.59 ± 1.93, *n* = 21; Table S4]. Furthermore, ant workers on the leaf-damaged plants were positioned more often near the leaf tip than those on the undamaged plants (Fig. 3; Table S4). Values of CV (coefficient of variation) in the relative positions of ant workers within a leaf did not differ between the treatments (Table S4).

### Nectar volume and sugar concentration

The nectar volume secreted from a leaf base EFN was approximately two times greater than that from an edge EFN [leaf base EFN, 0.14 ± 0.024 µl (mean ± SD), *n* = 20 and edge EFN, 0.064 ± 0.04 µl, *n* = 20; *t* test, *t* = 3.223, *P* < 0.001]. Sugar concentrations (mean ± SD) in the nectar secreted from the leaf base EFNs and edge EFNs were 177.8 ± 51.6 µg µl^−1^ and 183.8 ± 44.2 µg µl^−1^, respectively. These values did not significantly differ (*t* test, *t* = 0.395, *P* = 0.695).

### EFN utilization by ants

The nectar-sucking times of the two ant species (*P. noda* and *C. teranishii*) were similar (GLM, *P* = 0.21). Therefore, the data for these two species were pooled for comparing the nectar-sucking time from each EFN type. The time spent at the leaf base EFNs was longer than that along the edge EFNs [base EFNs, 33. 80 ± 109.14 s (mean ± SD), *n* = 20 and edge EFNs, 2.49 ± 1.24 s, *n* = 20; GLMM, Estimate = − 2.59, SE = 0.41, *z* = − 6.35, *P* < 0.001].

### Effects of two EFN types on ant attraction

**Fig. 4:**
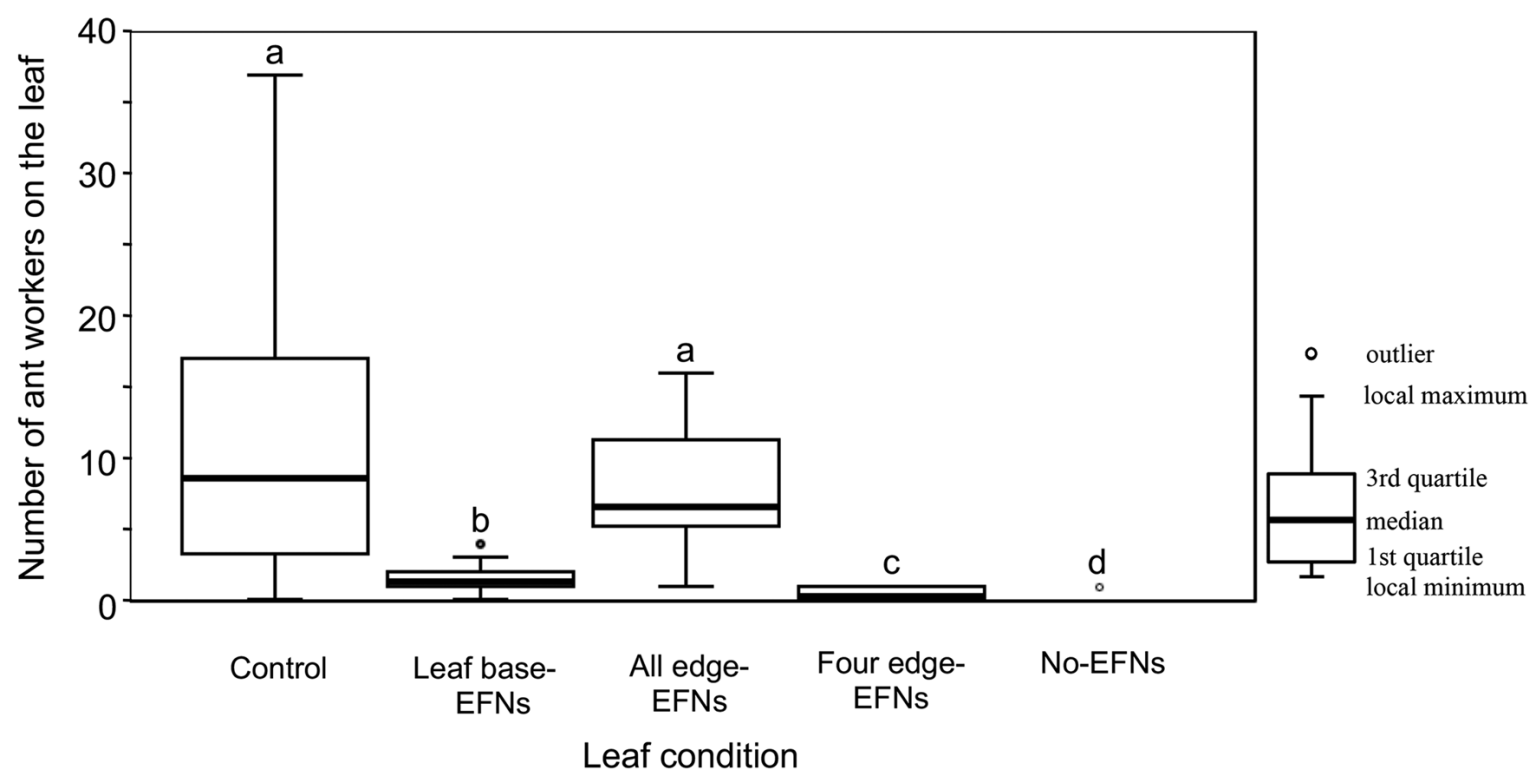
Numbers of ant workers on various EFN-treated leaves of *Mallotus japonicus*. In each treatment, several EFNs were covered with varnish to remove their functionality (refer to “Materials and methods” for details). For each treatment, *n* was 22 except for the no EFNs treatment (*n* = 10). Ant abundances differed among the treatments (GLM, *P* < 0.001). Different letters denote significant differences (Steel–Dwass test, *P* < 0.05)

**Fig. 5:**
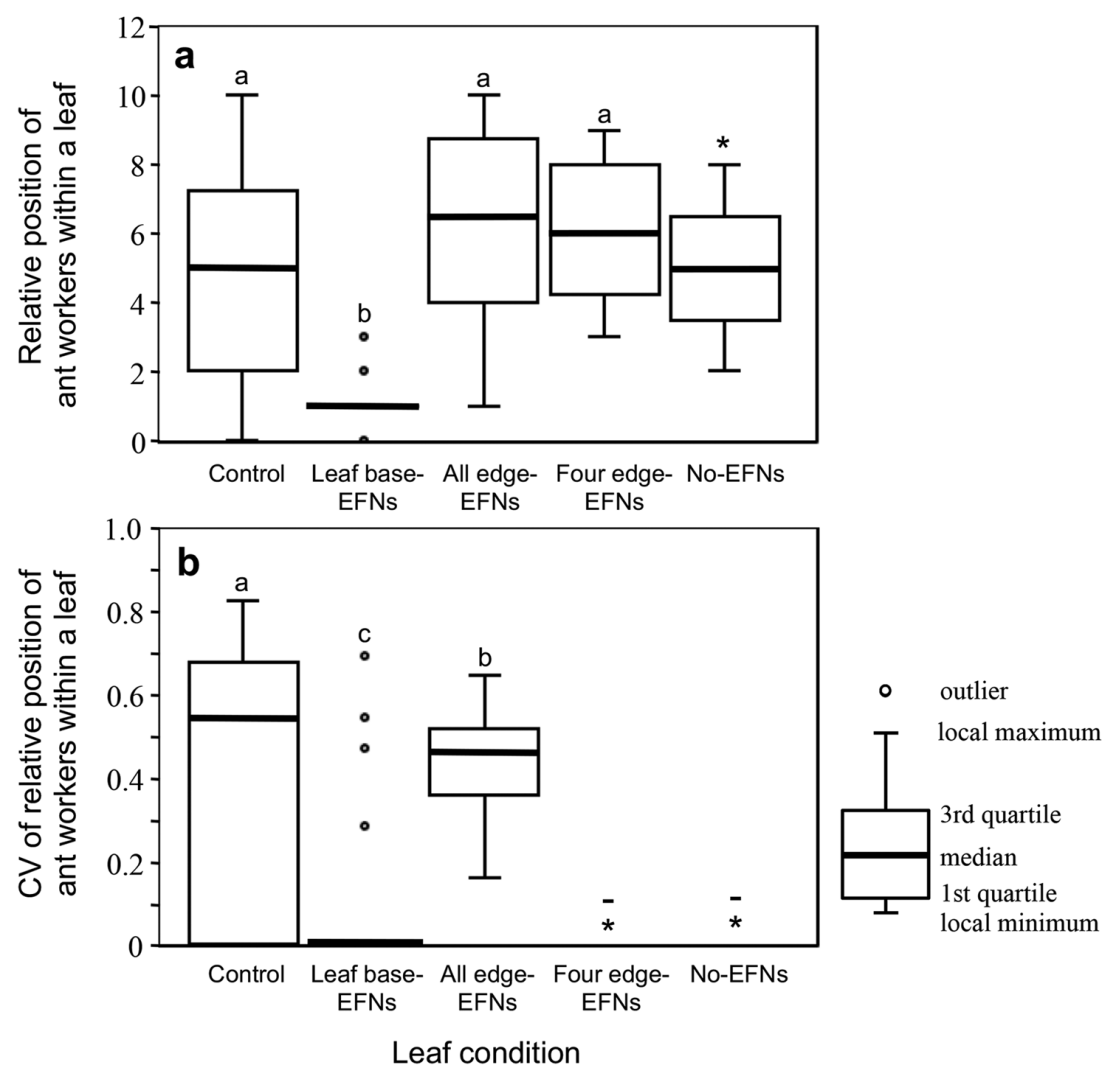
Relative position of ant workers (**a**), and the coefficient of variation (CV) of the relative position of ant workers (**b**), on a leaf of *Mallotus japonicus* under various EFN treatments. Control, *n* = 264; leaf base EFNs, *n* = 44; all edge EFNs, *n* = 158; four edge EFNs, *n* = 10; and no EFNs, *n* = 2. Both relative position and CV differed significantly among the treatments (GLM, *P* < 0.001). Different letters denote significant differences (Steel–Dwass test, *P* < 0.05). *Excluded from the analysis because of a lack of ant visits. – not calculated because of a lack of ant visits

On the 98 *M. japonicus* plants in the field, the following eight ant species were found: *P. noda* (20/98), *Pristomyrmex punctatus* Mayr (19/98), *C. teranishii* (14/98), *N. flavipes* (9/98), *Camponotus vitiosus* Smith (7/98), *C. osakensis* Forel (1/98), *Ochetellus glaber* Mayr (1/98), and *Formica japonica* Emery (1/98). The species composition of ants on the experimental leaves did not differ among the treatments (ANOSIM, global *R* = 1.04, *P* = 0.99). However, the numbers of ant workers on the leaves differed among the treatments (Table S5). Ants on the control leaves were most abundant, whereas ants on the no EFN leaves numbered the least. Ant abundances of the five treatments were rank ordered from largest to smallest as follows: control, all edge EFNs, leaf base EFNs, four edge EFNs, and no EFNs (Steel–Dwass test, *P* < 0.05; Fig. 4). Ants on leaf base EFN leaves were positioned more often near the leaf base than those on other treatment leaves (Steel–Dwass test, *P* < 0.05; Fig. 5a). The CV of positions on the control leaves was the largest, whereas that on the leaf base EFN leaves was the smallest (Steel–Dwass test, *P* < 0.05; Fig. 5b).

### Biotic defense by ants

Encounter rates of *S. litura* caterpillars and ant workers on the leaves of the EFN-covering treatments were as follows: control, 100% (22/22); leaf base EFNs, 22.7% (5/22); all edge EFNs, 100% (22/22); four edge EFNs, 40.9% (9/22); and no EFNs, 0% (0/10). The encounter rates on the leaves of the control and of all edge EFNs were higher than those of the leaf base EFNs (Fisher’s exact test, *P* < 0.001). The encounter rate on the four edge EFNs leaves was medium and did not significantly differ from those of the other treatments. The encounter rates of the herbivore with ants and ant abundance both increased with the number of ants (d.f. = 1, *P* < 0.001, *Estimate* = 0.69; Table S6), the relative position of ants (d.f. = 1, *P* = 0.007, *Estimate* = 1.15), and CV (d.f. = 1, *P* = 0.003, *Estimate* = 4.01).

Attack rates of ants against the herbivore in the control, leaf base EFNs, all edge EFNs, and four edge EFN treatments were, respectively, 50% (11/22), 40% (2/5), 45.5% (10/22), and 0% (0/9). The rate in the four edge EFN treatment significantly differed from those of other treatments (Fisher’s exact test, *P* < 0.001). The attack rates also increased with the number of ants (d.f. = 1, *P* < 0.001, *Estimate* = 0.38; Table S7) and the relative position of ants (d.f. = 1, *P* = 0.004, *Estimate* = 0.36). The CV did not significantly affect the attack rates (d.f. = 1, *P* = 0.069, *Estimate* = 1.70).

## Discussion

Regarding the functions of EFNs, the results demonstrate that the two types of EFNs in *M. japonicus* exhibit somewhat different effects on the plant’s ant partners. The leaf base EFNs play the fundamental role of ant attraction, whereas the role of the leaf edge EFNs is to extend the foraging area of ants from around the leaf base to the leaf tip, in addition to attracting ants. Leaf damage increased the number of edge EFNs in new leaves, a response by the plant that would increase ant abundance and would likely extend the total foraging area of ant workers on the plant. The present results support our hypothesis that two types of EFNs play different roles in biotic defense by ants in *M. japonicus*.

Our experiment revealed that both leaf base and edge EFNs could attract ant workers. The total volume of nectar secreted by all leaf edge EFNs is considered to be greater than that secreted by leaf base EFNs because leaf edge EFNs are more numerous. However, the nectar volume secreted by a single leaf base EFN is twice the amount secreted by a single leaf edge EFN. If the number of edge EFNs on a leaf is smaller than five, the ant-attractive ability of the leaf base EFNs should be greater than that of edge EFNs (leaf base EFNs vs. four edge EFNs in Fig. 4). Such a small number of leaf edge EFNs is often observed in the field (Yamawo et al. 2012b). The sugar concentrations in the nectars did not differ between the leaf base EFNs and edge EFNs. Therefore, the present results indicate that an intensive arrangement of nectar is desirable for ant attraction. EFN-bearing plants generally locate principal EFNs at their leaf base (Koptur 1992). Rios et al. (2008) have reported that herbivores feeding on EFN-bearing plants avoid the leaf base of fresh leaves when migrating from consumed leaves, largely because ants are present at the leaf base, indicating that the leaf base EFNs act to obstruct herbivore migration within a plant. Consequently, the EFNs at the leaf base would often escape herbivory. This is obviously advantageous for maintaining ant attraction in EFN-bearing plants.

In contrast, the leaf edge EFNs mainly exerted other effects on ants, such as increasing ant abundance on the leaf (Fig. 4) and broadening the ant foraging area from leaf base to tip (Fig. 5). In general, the efficacy of biotic defense by ants depends on the total number of ant workers on the plant (Ness 2003; Rios et al. 2008) because ant aggression toward insect herbivores increases with the number of workers nearby (Sakata and Katayama 2001). Moreover, the patrolling area of ants on the plant is an important factor for herbivore exclusion because, in many plants, ants deter herbivores through direct attack (de la Fuente and Marquis 1999). The scattered locations of edge EFNs should cause ants to walk widely around leaves. In the present study, the extension of ant foraging area on a leaf increased the likelihood of encounters between the ants and the herbivore, *S. litura*. This in turn increased the attack rates of ants on this herbivore (Fig. 5). Therefore, using edge EFNs, *M. japonicus* plants are capable of enhancing the efficacy of biotic defense by ants. In addition, ant presence on the leaf is considered sufficient to deter visits of lepidopteran herbivores to the plant because, in some cases, lepidopterans avoid oviposition altogether on the host plants that emit ant odors (Offenberg et al. 2004).

In leaf-damaged *M. japonicus*, the number of edge EFNs increased in the new leaves that developed after the leaf damage (Fig. 2), whereas the number of the leaf base EFNs was always the same, two per leaf. On these new leaves of leaf-damaged plants, more ant workers were attracted at the leaf tip than at the leaves of undamaged plants. Hence, the *M. japonicus* plant appears to influence the foraging area of ants by changing the number of edge EFNs available to them. When ant workers foraged near the leaf tip, the attack rate of ants on the herbivore increased. In this way, more edge EFNs obviously enhanced the efficacy of indirect defense by ants.

Increased EF nectar production increases ant activity and aggressiveness and is considered to favor mutualistic ants rather than parasitic ones (Bixenmann et al. 2011; Heil 2013). This partner choice by plants has been considered to be an effective mechanism for stabilizing ant–plant mutualisms (Bull and Rice 1991; Grasso et al. 2015). For *M. japonicus* plants in the field, most mutualistic ant species such as *Pheidole noda* and *Crematogaster teranishii* are more frequently observed on EF nectar-rich plants than on other plants, and non-aggressive ant species are observed to visit the plants having less EF-nectar (Yamawo et al. 2014, 2017). These observations suggest that the increased secretion of EF nectar can enable the function of partner choice in *M. japonicus* plants as well.

EFN-bearing plants generally have multiple types of EFNs, which differ in terms of size, position, and/or response to leaf damage (Delgado et al. 2017; Escalante-Pérez et al. 2012; Fahn 1987; Millán-Cañongo et al. 2014; O’Dowd 1979). Many of these plants have large EFNs on their leaf bases and small EFNs on their leaf edges (Baker et al. 1978; Delgado et al. 2017; Kowarik and Säumel 2007; Tilman 1978). The study results suggest that such variations in EFNs exert different effects on the behaviors of ant partners on plants such as *M. japonicus*.

## Electronic supplementary material

Below is the link to the electronic supplementary material.

Supplementary material 1 (PDF 139 kb)

## References

[CR1] Armbruster WS (1993). Evolution of plant pollination systems: hypotheses and tests with the neotropical vine *Dalechampia*. Evolution.

[CR2] Baker HG, Opler PA, Baker I (1978). A comparison of the amino acid complements of floral and extrafloral nectars. Bot Gaz.

[CR3] Bixenmann RJ, Coley PD, Kursar TA (2011). Is extrafloral nectar production induced by herbivores or ants in a tropical facultative ant-plant mutualism?. Oecologia.

[CR4] Bronstein JL (1998). The contribution of ant–plant protection studies to our understanding of mutualism. Biotropica.

[CR5] Bull JJ, Rice WR (1991). Distinguishing mechanisms for the evolution of co-operation. J Theor Biol.

[CR6] de la Fuente MAS, Marquis RJ (1999). The role of ant-tended extrafloral nectaries in the protection and benefit of a Neotropical rainforest tree. Oecologia.

[CR7] Delgado MN, Somavilla NS, Báo SN, Rossatto DR (2017). Testing the optimal defense hypothesis in *Stryphnodendron adstringens* (Fabaceae, Mimosoideae) leaves: the role of structure, number, position and nectar composition of extrafloral nectaries. Plant Spec Biol.

[CR8] Development R (2012). R: a language and environment for statistical computing.

[CR9] Doebeli M, Knowlton N (1998). The evolution of interspecific mutualisms. Proc Nat Acad Sci USA.

[CR10] Escalante-Pérez M, Jaborsky M, Lautner S, Fromm J, Müller T, Dittrich M, Kunert M, Boland W, Hedrich R, Ache P (2012). Poplar extrafloral nectaries: two types, two strategies of indirect defenses against herbivores. Plant Physiol.

[CR11] Fahn A (1987). The extrafloral nectaries of *Sambucus nigra*. Ann Bot.

[CR12] Ferriere R, Bronstein JL, Rinaldi S, Law R, Gauduchon M (2002). Cheating and the evolutionary stability of mutualisms. Proc R Soc Lond B.

[CR13] Grasso DA, Pandolfi C, Bazihizina N, Nocentini D, Nepi M, Mncuso S (2015). Extrafloral-nectar-based partner manipulation in plant-ant relationships. AoB Plants.

[CR14] Heil M (2013). Let the best one stay: screening of ant defenders by *Acacia* host plants functions independently of partner choice or host sanctions. J Ecol.

[CR16] Heil M, Barajas-Barron A, Orona-Tamayo D, Wielsch N, Svatos A (2014). Partner manipulation stabilises a horizontally transmitted mutualism. Ecol Lett.

[CR17] Koptur S, Bernays EA (1992). Extrafloral nectary-mediated interactions between insects and plants. Insect–plant interactions.

[CR18] Kowarik I, Säumel I (2007). Biological flora of Central Europe: *Ailanthus altissima* (Mill.) Swingle. Perspect Plant Ecol.

[CR19] Leigh EG (2010). The evolution of mutualism. J Evol Biol.

[CR20] Millán-Cañongo C, Orona-Tamayo D, Heil M (2014). Phloem sugar flux and jasmonic acid-responsive cell wall invertase control extrafloral nectar secretion in *Ricinus communis*. J Chem Ecol.

[CR21] Ness JH (2003). *Catalpa bignonioides* alters extrafloral nectar production after herbivory and attracts ant bodyguards. Oecologia.

[CR22] O’Dowd DJ (1979). Foliar nectar production and ant activity on a neotropical tree, *Ochroma pyramidale*. Oecologia.

[CR23] Offenberg J, Nielsen MG, Maclntosh DJ, Havanon S, Aksornkoae S (2004). Evidence that insect herbivores are deterred by ant pheromones. Proc R Soc Lond B.

[CR24] Pulice CE, Packer AA (2008). Simulated herbivory induces extrafloral nectary production in *Prunus avium*. Funct Ecol.

[CR25] Radhika V, Kost C, Bartram S, Heil M, Boland W (2008). Testing the optimal defence hypothesis for two indirect defences: extrafloral nectar and volatile organic compounds. Planta.

[CR26] Rios RS, Marquis RJ, Flunker JC (2008). Population variation in plant traits associated with ant attraction and herbivory in *Chamaecrista fasciculata* (Fabaceae). Oecologia.

[CR27] Sakata H, Katayama N (2001). Ant defence system: a mechanism organizing individual responses into efficient collective behavior. Ecol Res.

[CR28] Suzuki MF, Ohashi K (2014). How does a floral colour-changing species differ from its non-colour changing congener?—a comparison of trait combinations and their effects on pollination. Funct Ecol.

[CR29] Tilman D (1978). Cherries, ants and tent caterpillars: timing of nectar production in relation to susceptibility of caterpillars to ant predation. Ecology.

[CR30] Vander Wall SB (2010). How plants manipulate the scatter-hoarding behaviour of seed-dispersing animals. Philos Trans R Soc Lond B.

[CR31] Washitani I, Takenaka A (1987). Gap-detecting mechanism in the seed germination of *Mallotus japonicus* (Thunb.) Muell. Arg., a common pioneer tree of secondary succession in temperate Japan. Ecol Res.

[CR32] Wilder SM, Eubanks MD (2010). Extrafloral nectar content alters foraging preferences of a predatory ant. Biol Lett.

[CR33] Wooley SC, Donaldson JR, Gusse AC, Lindroth RL, Stevens MT (2007). Extrafloral nectaries in aspen (*Populus tremuloides*): heritable genetic variation and herbivore-induced expression. Ann Bot.

[CR34] Wright GA, Baker DD, Palmer MJ, Stabler D, Mustard JA, Power EF, Borland AM, Stevenson PC (2013). Caffeine in floral nectar enhances a pollinator’s memory of reward. Science.

[CR35] Yamawo A, Suzuki N (2018). Induction and relaxation of extrafloral nectaries in response to simulated herbivory in young *Mallotus japonicus* plants. J Plant Res.

[CR36] Yamawo A, Katayama N, Suzuki N, Hada Y (2012). Plasticity in the expression of direct and indirect defence traits of young plants of *Mallotus japonicus* in relation to soil nutritional conditions. Plant Ecol.

[CR37] Yamawo A, Suzuki N, Tagawa J, Hada Y (2012). Leaf ageing promotes the shift in defence tactics in *Mallotus japonicus* from direct to indirect defence. J Ecol.

[CR38] Yamawo A, Tagawa J, Hada Y, Suzuki N (2014). Different combinations of multiple defence traits in an extrafloral nectary-bearing plant growing under various habitat conditions. J Ecol.

[CR39] Yamawo A, Tokuda M, Katayama N, Yahara T, Tagawa J (2015). Ant-attendance in extrafloral nectar-bearing plants promotes growth and decreases the expression of traits related to direct defenses. Evol Biol.

[CR40] Yamawo A, Hada Y, Tagawa J (2017). Aggressiveness of ants attracted to the extrafloral nectary-bearing plant, *Mallotus japonicus*, and temporal fluctuations in their abundance. Entomol Sci.

