# Correction to: Extraforal nectary-bearing plant *Mallotus japonicus* uses diferent types of extraforal nectaries to establish efective defense by ants

**DOI:** 10.1007/s10265-020-01193-0

**Published:** 2020-04-25

**Authors:** Akira Yamawo, Nobuhiko Suzuki, Jun Tagawa

**Affiliations:** 1 Department of Applied Biological Sciences, Faculty of Agriculture, Saga University, Saga, 840-8502 Japan; 2 Department of Biosphere–Geosphere System Science, Faculty of Informatics, Okayama University of Science, Okayama, 700-0005 Japan

## Correction to: Journal of Plant Research (2019) 132:499–507 10.1007/s10265-019-01119-5

The article Extraforal nectary-bearing plant *Mallotus japonicus* uses diferent types of extraforal nectaries to establish efective defense by ants, written by Akira Yamawo, Nobuhiko Suzuki and Jun Tagawa, was originally published Online First without Open Access. After publication in volume 132, issue 4, page 499–507 the author decided to opt for Open Choice and to make the article an Open Access publication. Therefore, the copyright of the article has been changed to © The Author(s) 2020 and the article is forthwith distributed under the terms of the Creative Commons Attribution 4.0 International License (https://creativecommons.org/licenses/by/4.0/), which permits use, duplication, adaptation, distribution and reproduction in any medium or format, as long as you give appropriate credit to the original author(s) and the source, provide a link to the Creative Commons license, and indicate if changes were made.

The original article was updated.

