# Supplementary material for: Extrafloral nectary-bearing plant Mallotus japonicus uses different types of extrafloral nectaries to establish effective defense by ants
Source: J Plant Res. 2019 Jun 21;132(4):499–507. doi: 10.1007/s10265-019-01119-5 (PMC7196952; doi:10.1007/s10265-019-01119-5)
Supplement: Supplementary file 1 — Supplementary material 1 (PDF 139 kb) [file 10265_2019_1119_MOESM1_ESM.pdf]

Electronic supplementary materials

Title:

Extrafloral nectary-bearing plant *Mallotus japonicus* uses different types of extrafloral nectaries to establish effective defense by ants

Authors:

Akira Yamawo *[1], Nobuhiko Suzuki[1], Jun Tagawa[2]

Journal:

Journal of Plant Research

Correspondence:

Akira Yamawo

Contents:

Table S1-S7

Table S1. Results of GLM analyzing the effects of leaf damage treatment, types of EFNs, time (pre- or post-treatment), and their interactions

Factors	d.f.	Deviance	Residential d.f.	Residential deviance	*P*-value
Treatment (T)	1	25.57	176	311.07	< 0.01
EFN types (Ty)	1	101.75	175	209.32	< 0.01
Time (Ti)	1	21.9	174	187.42	< 0.01
T × Ty	1	7.66	173	179.76	< 0.01
T × Ti	1	11.91	172	167.84	< 0.01
Ty × Ti	1	9.77	171	158.07	< 0.01
T × Ty × Ti	1	5.83	170	152.24	0.016

Table S2. Results of GLM analyzing the effects of leaf damage treatment, time (pre- or post-treatment), and their interactions on the two types of EFNs

	Leaf base EFNs				Leaf edge EFNs			
Factors	Deviance	Residential d.f.	Residential deviance	*P*-value	Deviance	Residential d.f.	Residential deviance	*P*-value
Treatment (T)	0.006	85	1.467	0.938	17.39	88	128.27	< 0.01
Time (Ti)	0.006	84	1.462	0.940	17.31	87	110.96	< 0.01
T × Ti	0.006	83	1.456	0.938	10.46	86	80.5	< 0.01

Table S3. Results of GLM analyzing the pre- and post-treatment effects of leaf damage treatment on leaf edge EFNs

	Deviance	Residential d.f.	Residential deviance	*P*-value
Pre-treatment	0.09	43	33.5	0.76
Post-treatment	1.84	43	33.74	< 0.01

Table S4. Results of GLM analyzing the effects of leaf damage treatment on ant abundance, relative position of ants, and CV of ant position

	d.f.	Deviance	Residential d.f.	Residential deviance	*P*-value
Number of ants	1	27.57	43	33.04	< 0.01
Relative position of ants	1	62.41	41	17.39	< 0.01
CV of ant position	1	1.15	43	23.1	0.283

Table S5. Results of GLM analyzing the effects of EFN-covering treatment on ant abundance, relative position of ants, and CV of ant position

	d.f.	Deviance	Residential d.f.	Residential deviance	*P*-value
Number of ants	4	233.46	105	93.72	< 0.01
Relative position of ants	4	187.74	105	91.3	< 0.01
CV of ant position	2	6.43	63	16.72	0.039

Table S6. Relations between the encounter rates of the herbivore with ants and ant abundance, relative position of ants, or CV of ant position

	Estimate	SE	Z-value	*P*-value
Number of ants	0.693	0.177	3.915	< 0.001
Relative position of ants	1.151	0.332	3.471	< 0.001
CV of ant position	4.007	1.329	3.015	0.003

Table S7. Relations between the attack rates of ants against the herbivore and ant abundance, relative position of ants, or CV of ant position

	Estimate	SE	Z-value	*P*-value
Number of ants	0.381	0.082	4.621	< 0.001
Relative position of ants	0.362	0.126	2.88	0.004
CV of ant position	1.699	0.936	1.815	0.069
